# Comparison of postoperative complications between segmentectomy and lobectomy by video-assisted thoracic surgery: a multicenter study

**DOI:** 10.1186/s13019-019-1021-9

**Published:** 2019-11-07

**Authors:** Benoît Bédat, Etienne Abdelnour-Berchtold, Thomas Perneger, Marc-Joseph Licker, Alexandra Stefani, Matthieu Krull, Jean Yannis Perentes, Thorsten Krueger, Frédéric Triponez, Wolfram Karenovics, Michel Gonzalez

**Affiliations:** 1 Division of Thoracic and Endocrine Surgery, University Hospitals of Geneva, Geneva, Switzerland; 2 Division of Thoracic Surgery, Centre Hospitalier Universitaire Vaudois, Lausanne, Switzerland; 3 Division of Clinical Epidemiology, University Hospitals of Geneva, Geneva, Switzerland; 4 Division of Anesthesiology, University Hospitals of Geneva, Geneva, Switzerland

**Keywords:** Complication, Segmentectomy, Lobectomy, Video-assisted thoracic surgery

## Abstract

**Background:**

Compared to lobectomy by video-assisted thoracic surgery (VATS), segmentectomy by VATS has a potential higher risk of postoperative atelectasis and air leakage. We compared postoperative complications between these two procedures, and analyzed their risk factors.

**Methods:**

We reviewed the records of all patients who underwent anatomical pulmonary resections by VATS from January 2014 to March 2018 in two Swiss university hospitals. All complications were reported. A logistic regression model was used to compare the risks of complications for the two interventions. Adjustment for patient characteristics was performed using a propensity score, and by including risk factors separately.

**Results:**

Among 690 patients reviewed, the major indication for lung resection was primary lung cancer (86.4%) followed by metastasis resection (5.8%), benign lesion (3.9%), infection (3.2%) and emphysema (0.7%). Postoperatively, there were 80 instances (33.3%) of complications in 240 segmentectomies, and 171 instances (38.0%) of complications in 450 lobectomies (*P* = 0.73). After adjustment for the patient’s propensity to be treated by segmentectomy rather than lobectomy, the risks of a complication remained comparable for the two techniques (odds ratio for segmentectomy 0.91 (0.61–1.30), *p* = 0.59). Length of hospital stay and drainage duration were shorter after segmentectomy. On multivariate analysis, an American Society of Anesthesiologists score above 2 and a forced expiratory volume in one second below 80% of predicted value were significantly associated with the occurrence of complications.

**Conclusions:**

The rate of complications and their grade were similar between segmentectomy and lobectomy by VATS.

## Background

Currently, pulmonary lobectomy is routinely performed by video-assisted thoracic surgery (VATS) for several indications. In patients with lung cancer, VATS lobectomy is considered safe and effective in early stage non-small cell lung cancer (NSCLC) [1–3]. Furthermore, compared to open lobectomy, the VATS approach is associated with less postoperative pain and better quality of life [4]. In parallel, VATS segmentectomy is increasingly proposed as an alternative to lobectomy to spare pulmonary parenchyma for benign and malignant lesions. In patients with NSCLC, segmentectomy seems to ensure equivalent oncological outcomes for tumor sizes smaller than 2 cm without nodal involvement [5, 6].

Evidence of functional benefit of segmentectomy over lobectomy remains low with debatable results, but the former seems to better preserve lung function via an increased function of the ipsilateral non-operated lobe [7, 8]. However, the segmentectomy procedure requires more extensive and deeper dissection into the hilum and division of the intersegmental plane, which may lead to an higher rate of prolonged air leaks [9]. Furthermore, the use of staplers to divide the intersegmental plane can induce compression of the adjacent lung and may cause atelectasis and pneumonia of the remnant lobe. Few studies that reported incidence and severity of postoperative complications following segmentectomy and lobectomy by VATS, and other retrospective studies that have yielded contradictory results and had study limitations (e.g., small sample or single-center cohort; incomplete data about complications; analysis of patients with early-stage cancer; lack of analysis of predictive factors for complications) [9–13].

The aim of this study was to compare the incidence of post-operative complications between VATS segmentectomy and VATS lobectomy and to identify their predictive factors.

## Methods

### Patients

We reviewed the records of all patients who underwent anatomical pulmonary resections by VATS from January 2014 to March 2018 at the University Hospitals of Lausanne and Geneva in Switzerland. The local ethics committee approved this study (referral number: 2018–00179) and waived the need to obtain informed patient consent. All indications were included. All patients with NSCLC were presented at an interdisciplinary tumour board. The indication for VATS lobectomy for lung cancer was proposed in patients with tumors resectable by a minimally invasive approach. The indication for VATS segmentectomy for lung cancer was proposed in patients with a nodule smaller than 2 cm without nodal involvement. VATS segmentectomy have been performed “intentionally”, unrelated to the fitness of the patient.

### Surgical technique

Four surgeons who had each performed > 100 cases of VATS lobectomy included in this study carried out all segmentectomies. Surgical resections were undertaken using an anterior three-port approach. All vascular structures were transected using endoscopic staplers or an energy device and complete dissection of hilar and mediastinal lymph nodes was carried out in patients with NSCLC. All bronchial structures were transected using endoscopic staplers. In segmentectomy, the intersegmental plane was divided using staplers. In lobectomy, a fissure-less technique was performed preferentially.

### Classification of postoperative complications

Patient records were extracted from the hospital data management system. The following data were obtained: patient demographics and comorbidities; preoperative lung function; indication for surgery; type of pulmonary resection; duration of surgery; histologic findings; duration of drainage; length of hospital stay (LOS); postoperative complications and mortality.

The primary endpoint was the occurrence of the following cardiopulmonary complications: atrial fibrillation; acute myocardial ischemia; pneumothorax; hemothorax; prolonged air leak (PAL), defined as an air leak lasting beyond postoperative day 7; acute respiratory distress syndrome (ARDS), defined using the Berlin classification [14]; pneumonia, defined by the need for antibiotics according to the appearance of new lung infiltrate at chest-X rays, fever, or an elevated white blood cell count > 12,000 per ml; acute pulmonary edema; massive subcutaneous emphysema; atelectasis; pulmonary embolism; chylothorax; cardiac infarction; empyema; bronchopulmonary fistula.

The secondary endpoint was the occurrence of any other complication, such as recurrent and phrenic nerve injury, gastrointestinal ileus, colitis, gastroparesis, upper gastrointestinal bleeding, septic shock and acute renal failure.

The complications were then classified in 3 groups according to the Thoracic Morbidity and Mortality system: [15] Minor complications (grade I and II), major complications (grade III and IV) and mortality (grade V). For patients experiencing more than one complication, the most severe grade was assigned.

### Statistical analysis

For patient characteristics, chi-squared or Fischer’s exact tests were used to analyze categorical variables. A T-test or Mann-Whitney U test was used to analyze continuous variables.

Since patients were assigned to lobectomy or segmentectomy based on their clinical characteristics (and not at random), the comparison of complication rates required adjustment for potential confounders. A propensity score was calculated from a logistic regression model, including following variables: gender, the presence of chronic obstructive pulmonary disease (COPD), indication for surgery (primary lung cancer, metastasis, benign lesion, emphysema, infection), and location of the lesion (lobe). This model represented the probability of being assigned to VATS segmentectomy as opposed to VATS lobectomy. A Nagelkerke R [2] test was used to measure the goodness-of-fit and the area under the curve was used to measure propensity-score performance. A logistic regression analysis was used with the propensity-score model and the type of intervention to assess the occurrence of complications. We compared the distributions of propensity scores in patients treated with segmentectomy and lobectomy, and excluded tail distributions that received only one treatment modality (49 patients with propensity < 0.10 were all treated with lobectomy, and 6 patients with propensity > 0.85 were all treated with segmentectomy). We then obtained a classic logistic regression model where the occurrence of any complication was a function of treatment modality and of the continuous propensity score (Model 1). To verify the robustness of the result we also performed a conditional logistic regression analysis, using 15 matched sets of patients (propensity 0.100–0.149, 0.150–0.199, 0.200–0.249, etc) (Model 2). These analyses were repeated for cardio-pulmonary complications only.

For the occurrence of any complication, the predictive factors for complications were also analyzed using a multivariate logistic regression models. The cutoffs of the forced expiratory volume in one second (FEV1) and the diffusing capacity of the lungs for carbon monoxide (DLCO) predictive values were chosen according to established recommendations [16].

## Results

### Patients and surgery

Patients’ demographic and clinical characteristics are summarized in Table 1. Overall, 690 patients were reviewed, including 240 who had VATS segmentectomies and 450 who had VATS lobectomies. The conversion rate to thoracotomy was 5.8% for both procedures. Most interventions were performed in center 1 (62.6%). As shown in Table 1, preoperative patient characteristics were not significatively different regardless the type of surgery for age, comorbidities and American Society of Anesthesiologists (ASA) score. The major indication for lung resection was primary lung cancer (86.4%) followed by metastasis resection (5.8%), benign lesion (3.9%), infection (3.2%) and emphysema (0.7%). The type of surgery differed among indications (Table 1). Patients with primary lung cancer were more likely to have VATS lobectomy than VATS segmentectomy (91.1% versus 77.5%, respectively). On the other hand, resection of metastasis and benign lesions was likely performed by VATS segmentectomy. The location of the lung resection differed significantly between the procedures, with an upper right location predominantly found in the lobectomy group and a left location in the segmentectomy group. Overall, a systematic lymph node dissection was significantly more often performed during VATS lobectomy (Table 1). The FEV1 and DLCO did not differ between procedures.

**Table 1:** Correlations of patient characteristics with type of intervention by VATS

Characteristics	N (%)	Segmentectomy *N* = 240	Lobectomy *N* = 450	P
Center				
1	432 (62.6)	143 (59.6)	289 (64.2)	0.23
2	258 (37.4)	97 (40.4)	161 (35.8)	
Gender, man	375 (54.3)	116 (48.3)	259 (57.6)	0.02
Age				
21–59	168 (24.3)	59 (24.6)	109 (24.2)	0.96
60–69	248 (35.9)	89 (37.1)	159 (35.3)	
70–79	215 (31.2)	72 (30.0)	143 (31.8)	
80–90	59 (8.6)	20 (8.3)	39 (8.7)	
ASA score				
1	6 (0.9)	2 (0.8)	4 (0.9)	0.77
2	377 (54.6)	134 (56.1)	243 (54.4)	
3	295 (42.8)	99 (41.4)	196 (43.8)	
4	8 (1.2)	4 (1.7)	4 (0.9)	
BMI (mg/kg^2^)				
14.5–18.4	46 (6.7)	17 (7.1)	29 (6.5)	0.46
18.5–24.9	305 (44.2)	101 (42.2)	204 (45.7)	
25–29.9	223 (32.3)	86 (36.1)	137 (30.7)	
30.0–57.0	110 (15.9)	34 (14.3)	76 (17.0)	
Active smoker	293 (42.5)	91 (37.9)	202 (45.0)	0.07
PY, mean (SD)	35.4 (30)	34.2 (29.6)	36.1 (30.2)	0.46
Arterial hypertension	345 (48.6)	121 (50.4)	214 (47.6)	0.47
Cardiopathy	88 (12.8)	26 (10.8)	62 (13.8)	0.27
Diabetes	102 (14.8)	31 (12.9)	71 (15.8)	0.30
COPD	259 (37.5)	99 (41.3)	160 (35.6)	0.14
History of cancer	229 (33.2)	93 (38.8)	136 (30.2)	0.02
FEV1 (%), 28 missing				
24–59	53 (8.0)	21 (9.1)	32 (7.4)	0.42
60–79	169 (25.5)	64 (27.7)	105 (24.4)	
80–170	440 (66.5)	146 (63.2)	294 (68.2)	
DLCO (%), 54 missing				
26–60	125 (19.7)	49 (22.3)	76 (18.3)	0.48
61–80	238 (37.4)	79 (35.9)	159 (38.2)	
81–154	273 (42.9)	92 (41.8)	181 (43.5)	
Indication				
Lung cancer (primary)	596 (86.4)	186 (77.5)	410 (91.1)	< 0.001
Metastasis	40 (5.8)	27 (11.3)	13 (2.9)	
Benign lesion	27 (3.9)	18 (7.5)	9 (2.0)	
Emphysema	5 (0.7)	0 (0)	5 (1.1)	
Infection	22 (3.2)	9 (3.8)	13 (2.9)	
Side, right	375 (54.3)	89 (37.1)	286 (63.6)	< 0.001
Location (lobe)				
Upper right	198 (28.7)	41 (17.1)	157 (34.9)	< 0.001
Middle right	38 (5.5)	2 (0.8)	36 (8.0)	
Lower right	128 (18.6)	46 (19.2)	82 (18.2)	
2 right lobes	11 (1.6)	0 (0)	11 (2.4)	
Upper left	180 (26.1)	76 (31.7)	104 (23.1)	
Lower left	135 (19.6)	75 (31.3)	60 (13.3)	
SLND	614 (89.0)	203 (84.6)	411 (91.3)	0.01

*VATS* Video-assisted thoracic surgery, *ASA* American Society of Anesthesiologists score, *BMI* Body mass index, *PY* pack-year, *COPD* Chronic obstructive pulmonary disease, *FEV1* Forced expiratory volume in one second, *DLCO* Diffusing capacity of the lungs for carbon monoxide, *SLND* systematic lymph node dissection

As shown in Table 2, in patients with primary lung cancer, larger tumor and nodal involvement were strongly associated with VATS lobectomy. The duration of operation and the rate of conversion to thoracotomy were similar between both groups.

**Table 2:** Procedures and postoperative outcomes

Outcomes	Segmentectomy *N* = 240	Lobectomy *N* = 450	P
Patients with cardiopulmonary complication, N (%)	75 (31.3)	160 (35.6)	0.26
Patients with any complication, N (%)	80 (33.3)	171 (38.0)	0.22
Complication per patient if occurs, mean (SD)	1.65 (1.04)	1.67 (1.03)	0.87
TMM, N (%)			
Grade 1–2	61 (25.4)	131 (29.1)	0.73
Grade 3–4	17 (7.1)	38 (8.4)	
Grade 5	2 (0.8)	2 (0.4)	
Type of complications, N (%)			
BPN	30 (12.5)	67 (14.9)	0.42
PAL	21 (8.8)	51 (11.3)	0.36
FA	13 (5.4)	39 (8.7)	0.13
Pneumothorax	16 (6.7)	21 (4.7)	0.29
Hemothorax	3 (1.2)	9 (2)	0.56
ARDS	4 (1.7)	4 (0.9)	0.46
Atelectasia	5 (2.1)	14 (3.1)	0.63
Acute pulmonary edema	1 (0.4)	1 (0.2)	1.0
Massive subcutaneous emphysema	11 (4.6)	21 (4.7)	1.0
Chylothorax	0 (0)	1 (0.2)	1.0
Pulmonary embolism	2 (0.8)	1 (0.2)	0.28
BPF	0 (0)	1 (0.2)	1.0
Empyema	4 (1.7)	2 (0.4)	0.19
Cardiac infarction	0 (0)	3 (0.7)	0.55
Recurrent nerve paralysis	0 (0)	5 (1.1)	0.17
Acute renal failure	5 (2.1)	17 (3.8)	0.26
Septic shock	3 (1.2)	6 (1.3)	1.0
Cardiorespiratory arrest	2 (0.8)	0 (0)	0.12
Ileus	3 (1.2)	2 (0.4)	0.35
Upper GI bleeding	2 (0.8)	0 (0)	0.12
Gastroparesis	1 (0.4)	0 (0)	0.35
Reoperation	6 (2.5)	21 (4.7)	0.22
Duration of surgery, mean (SD)	147.1 (55.2)	150.3 (50.6)	0.44
Conversion	14 (5.8)	26 (5.8)	0.98
Oncological histology, N (%)			
NSCLC	170 (90.9)	359 (87.1)	0.055
SCLC	2 (1.1)	7 (1.7)	
NET (AC, TC, LNEC)	15 (8)	33 (8)	
Other	0 (0)	13 (3.2)	
Tumor size (mm), mean (SD)	17.5 (8.1)	30.7 (17.1)	< 0.001
Lymph node involvement	*N* = 183	*N* = 406	0.001
pN1	6 (3.3)	39 (9.6)	
pN2	10 (5.5)	47 (11.6)	
Drainage duration, median **[**IQR**]**	2 [1–3]	3 [1–5]	0.012
LOS, median **[**IQR**]**	6 [4–9]	7 [5–11]	0.045

*SD* Standard deviation, *NSCLC* Non-small cell lung cancer, *SCLC* Small-cell lung cancer, *NET* Neuroendocrine tumor, *TC* Typical carcinoid, *AC* Atypical carcinoid, *LNEC* Large cell neuroendocrine carcinoma, *LOS* Length of hospital stay, *TMM* Thoracic morbidity and mortality score, *BPN* Bronchopneumonia, *PAL* Prolonged air leak, *AF* Atrial fibrillation, *ARDS* Acute respiratory distress syndrome, *BPF* Bronchopulmonary fistula, *GI* gastrointestinal

### Complications

As shown in Table 2, overall, 33.3% of patients experienced postoperative complications after VATS segmentectomies and 38% after VATS lobectomies. Most patients had only one complication and the average number of complications per patient was not significantly different between segmentectomy and lobectomy. The reoperation rate was not significantly different for the two procedures. VATS sleeve lobectomy was performed in 14 patients, 50% of whom had cardiopulmonary complications. Upper bilobectomy was performed in 11 patients, 54% of whom had cardiopulmonary complications. According to the TMM system, the grade of complications was comparable between both procedures (Table 2). After VATS segmentectomy and VATS lobectomy, minor complications occurred in 25.4 and 29.1% of patients, respectively, major complications in 7.1 and 8.4% of patients, and death in 0.8 and 0.4% of patients. Furthermore, the type of procedure was not associated with any specific complication, especially prolonged air leak, atelectasis or bronchopneumonia (Table 2). The results were similar when we analyzed complications in patients with lung cancer (data not shown).

The logistic regression model to construct the propensity score is shown in Additional file 1 and included gender, presence of COPD, histology, and location of the resection. The comparison of propensity score between interventions is shown in Additional file 2, with a favorable AUC of 0.744 (CI 95% 0.706–0.782). According to this propensity score, we demonstrated that the occurrence of any complication or cardiopulmonary complication was similar between VATS segmentectomy and VATS lobectomy (Table 3).

**Table 3:** Logistic regression models for the occurrence of any complication, and of a cardiopulmonary complication

Models		Any complication		Cardiopulmonary complication	
		OR (95% CI)	P	OR (95% CI)	P
1	VATS segmentectomy^a^	0.91 (0.61–1.30)	0.58	0.93 (0.65–1.34)	0.71
	Propensity score^b^	0.42 (0.16–1.17)	0.10	0.46 (0.19–1.13)	0.049
2	VATS segmentectomy^a^	0.93 (0.70–1.24)	0.62	0.86 (0.60–1.24)	0.41

Model 1: standard logistic regression, where the propensity score was left as a continuous probability between 0 and 1. Model 2: Conditional logistic regression, with 15 sets matched for propensity (0.100–0.149, 0.150–0.199, 0.200–0.249, etc). Fifty-five patients with a propensity for segmentectomy < 0.10 and > 0.85 were excluded, as all were treated with the same intervention. ^a^ vs lobectomy

^b^ probability of 1 vs 0

*VATS* Video-assisted thoracic surgery, *OR* Odds ratio, *CI* Confidence interval

### Risk factors

The associations between patient characteristics and complications are shown in Additional file 3. The risk of complications increased significantly with age and then decreased in the oldest patients (41.4% in patients between 70 and 79 years old and 25.4% in patients between 80 and 90 years old). Compared to those with a normal body mass index, underweight patients had an almost 10% increased risk of complication. However, overweight or obesity did not seem to impact the risk of complications. The occurrence of complications was significantly associated with ASA score > 2, an increased number of pack years, presence of COPD, a decreased FEV1, and a decreased DLCO. The risk of complication also depended on the surgical indication, with a lower risk in patients with metastasis or benign lesion resection and a higher risk in patients with lung volume reduction surgery. However, while the size of the lesion differed between the two groups, size was unrelated to the occurrence of complications (mean 26.1 ± 17.2 without complication, 26.9 ± 16.8 with complication, *p* = 0.55).

In the multivariate analysis, an ASA score above 2, and a FEV1 below 80% were significantly associated with the occurrence of complications (Table 4). However, benign nodule and metastasis resection, as well as middle lobe resection were significantly associated with a better postoperative outcome (Table 4). The ASA score and the FEV1 were not correlated with TMM grade.

**Table 4:** Multivariate model of risk factors for complications (intervention type was forced into the model)

Risk factors	OR (95% CI)	P
Segmentectomy (vs lobectomy) by VATS	0.89 (0.62–1.28)	0.74
Center 2 (vs Center 1)	0.71 (0.50–1.01)	0.051
ASA 3–4 (vs 1–2)	1.55 (1.10–2.19)	0.011
FEV1 < 80% (vs **≥**80%)	1.61 (1.11–2.30)	0.009
Indication		0.042
Lung cancer (primary)	1 (reference)	–
Metastasis	0.32 (0.13–0.80)	0.015
Benign lesion	0.27 (0.08–0.95)	0.042
Emphysema	1.33 (0.21–8.31)	0.76
Infection	0.74 (0.22–2.44)	0.64
Location (lobe)		0.066
Upper (right or left)	1 (reference)	–
Middle right	0.32 (0.13–0.82)	0.018
Lower (right or left)	0.96 (0.68–1.36)	0.82
2 right lobes	2.15 (0.58–8.01)	0.25

*OR* Odds ratio, *CI* Confidence interval, *VATS* Video-assisted thoracic surgery, *ASA* American Society of Anesthesiologists score, *FEV1* Forced expiratory volume in one second

### Length of hospital stay

Segmentectomy by VATS was associated with a shorter duration of drainage than lobectomy by VATS (3 days, IQR [1–5] versus 2 days, IQR [1–3], respectively, *P* = 0.011) and a shorter length of hospital stay (7 days, IQR [5–11] versus 6 days, IQR [4–9], respectively, *P* = 0.045). The length of hospital stay (LOS) increased with the presence of complication (6.1 ± 3.5 days versus 13.4 ± 11 days, *P* < 0.001). The LOS was also affected by TMM degree of complication, with an average of 12.3 ± 8.4 days for patients with minor complications and 17.2 ± 16.3 days for patients with major complications (*P* = 0.0034).

## Discussion

Although complications after lobectomy have been characterized extensively, remarkably little is known about complications after anatomical resections by VATS. In this multicenter study, which compared complications between intentional segmentectomy and lobectomy by VATS, we have shown that: (i) the risk of complication after VATS segmentectomy was not significantly different than that after VATS lobectomy; (ii) the TMM scores were comparable between the two procedures; (iii) a FEV1 below 80% and an ASA score of 3–4 were associated with an increased risk of complications (regardless of the procedure); (iv) benign nodule and metastasis resection, as well as middle lobe resection were associated with a better postoperative outcome; and (v) the drainage duration and the LOS were shorter in the VATS segmentectomy group.

In our study, we have shown that the rate, the type and the grade of complication were not different between segmentectomy and lobectomy by VATS. Neither the dissection of the intersegmental plane nor the parenchymal compression during segmentectomy seem to increase the risk of pulmonary complications. A few retrospective studies compared postoperative complication rate after VATS anatomical resections and found contradictory results [9–13]. Our finding of no difference between segmentectomy and lobectomy in incidence of cardiopulmonary complications contrasts with the findings of Deng et al. [9] This study shown a higher incidence of PAL and pulmonary complications after VATS segmentectomy. Because of their small sample size in the segmentectomy group (*N* = 35), the authors suggested that a learning curve could explain this result. In their comparison of postoperative complications between segmentectomy (*N* = 39) and lobectomy (*N* = 81), Zhong et al. [12] found that despite the small sample size, postoperative complication rates were low and similar (12.8% vs. 12.3%, *P* = 0.94) and the most frequent complication was atrial fibrillation (2.6% vs. 3.7%). However, the patients had few comorbidities and only 18.9% of patients suffered from COPD. A study by Whang et al. [11] that included only pulmonary complications in a match-paired study of 94 segmentectomies and 94 lobectomies reported comparable postoperative pulmonary complication rates of 10.6% for segmentectomies and 17.2% for lobectomies (*P* = 0.1); 18% of patients suffered from COPD. Recently, Song et al. reported similar rates of postoperative pulmonary complications (15.3%) between segmentectomy (*N* = 41) and lobectomy (*N* = 122) [10]. Finally, Lin et al. compared segmentectomy (*N* = 32) and lobectomy (*N* = 47) by a single-port approach, finding no difference in the postoperative complication rate (*P* = 0.19) [13].

Although these studies showed similar results to our findings, their complication rates appeared lower. However, they included only patients with early-stage NSCLC and reported only cardio-pulmonary complications. One explanation for the higher rate of complications in our study is that we included all types of complications. We report postoperative complications routinely and extensively, which could increase the number of complications [17, 18]. In addition, the higher rate of patient comorbidities in our study, as compared to some other studies, could increase the rate of complication, as established in the European risk models for morbidity (Eurolung1) score [19]. In this sense, Deng et al. reported a higher rate of complications after VATS segmentectomy and lobectomy (68.6% vs. 74.1%), with a comorbidity rate comparable to our findings. Finally, our higher incidence of pneumonia in particular could be explained by the higher rate of comorbidities and patients with COPD.

In our study, predictive factors for complications include an ASA score > 2 and a FEV1 < 80% of the predicted value, regardless of the procedure. However, the ASA score and the FEV1 were not associated with the TMM grade. The ASA score as a predictive factor for complication was previously established in the Eurolung 1 score [19]. In thoracotomy patients, FEV1 < 60% has been associated with a higher rate of pulmonary complications [20, 21]. We also found that FEV1 < 60% was a predictive factor of post-operative complications in anatomical resection by VATS. As compared to an FEV1 above 80%, the risk of complication increased by 60% in patients with an FEV1 under 60% of the predicted value. Contrary to FEV1, a lower DLCO is not associated with an increased risk of complication. Similarly to our findings, Benattia et al. showed that the FEV1, but not DLCO, independently predicted postoperative outcome in VATS anatomical resection [22]. However, another study did not report FEV1 and DLCO as independent predictor factors for pulmonary complications after lobectomy by VATS [23]. According to our results, probably that preoperative risk assessment recommended by the ERS/ESTS task force to predict postoperative morbidity after resection by thoracotomy could be used for all anatomical resections by VATS. However, further studies are needed to confirm this hypothesis.

The postoperative complications after anatomical resection for other indications than NSCLC have not been studied yet. In our study, resection of metastasis and benign lesion are associated with a lower risk of postoperative complication, regardless of the type of resection. A less extensive dissection during intervention in these indications could explain our results.

Interestingly, the drainage duration and the LOS was shorter in VATS segmentectomy than in VATS lobectomy. Furthermore, VATS segmentectomy showed a similar rate of conversion as VATS lobectomy and could be considered safe. Further studies are needed to compare the global costs between the two procedures.

Our study had several limitations. First, it was a retrospective analysis of two centers experience in VATS segmentectomy and lobectomy and may introduce selection and information bias. Second, the low granularity of data regarding the management of complications allowed only a rough classification of the TMM grade. Third, post-operative management was based on local practice, which differed between the two centers, although both had similar proportions of VATS segmentectomies and VATS lobectomies. Fourth, data about VATS segmentectomies included the learning phase of the technique, which could have influenced the postoperative outcomes during the initial phase. We have not analyzed the difference in complication rates among the study periods. Fifth, as expected with registered data, several factors were missing from our data sets, including the 90-day mortality and comorbidity scores. For these reasons, the EuroLung1 EuroLung2 scores could not be used [19]. Sixth, perioperative data was lacking regarding the complexity of the procedure and perioperative complications. However, the large number of patients with VATS segmentectomy available for analysis, and the high granularity of data collected regarding complications are major strengths of this study. Finally, another limitation is that if the choice of treatment modality (segmentectomy vs lobectomy) was driven by unmeasured patient characteristics that are associated with the risk of complication, substantial residual confounding would presist (confounding by indication). Only a randomized trial comparing the two techniques would eliminate this potential problem.

## Conclusions

In conclusion, we did not observe significant differences in terms of postoperative complications between intentional VATS segmentectomies and lobectomies, even after adjustment for differences in patient characteristics. Both approaches seem to yield similar rates of complications and the occurrence of complications depends on the ASA score and the percentage of the predicted FEV1.

## Supplementary information

**Additional file 1.** Multivariate logistic regression models to predict segmentectomy by VATS (versus lobectomy by VATS) VATS: Video-assisted thoracic surgery, OR: Odds ratio, CI: Confidence interval, COPD: Chronic obstructive pulmonary disease.

**Additional file 2.** Comparison of propensity score according to type of intervention by VATS VATS: Video-assisted thoracic surgery, SD: Standard deviation, AUC: Area under the curve, CI: Confidence interval.

**Additional file 3.** Associations of patient characteristics with complications. VATS: Video-assisted thoracic surgery, ASA: American Society of Anesthesiologists Score, BMI: Body mass index, PY: pack-years, COPD: Chronic obstructive pulmonary disease, FEV1: Forced expiratory volume in one second, DLCO: Diffusing capacity of the lungs for carbon monoxide, SLND: systematic lymph node dissection.

## Data Availability

The datasets used and/or analysed during the current study are available from the corresponding author on reasonable request.

## Abbreviations

ARDS: Acute respiratory distress syndrome
ASA: American Society of Anesthesiologists
BPN: Bronchopneumonia
COPD: chronic obstructive pulmonary disease
DLCO: Diffusing capacity of the lungs for carbon monoxide
FEV1: Forced expiratory volume in one second
LOS: Length of hospital stay
NSCLC: non-small cell lung lancer
PAL: Prolonged air leak
PY: pack-year
TMM: Thoracic morbidity and mortality score
VATS: video-assisted thoracic surgery
