# Supplementary material for: Comparison of postoperative complications between segmentectomy and lobectomy by video-assisted thoracic surgery: a multicenter study
Source: J Cardiothorac Surg. 2019 Nov 7;14:189. doi: 10.1186/s13019-019-1021-9 (PMC6836384; doi:10.1186/s13019-019-1021-9)
Supplement: Supplementary file 1 — Additional file 1. Multivariate logistic regression models to predict segmentectomy by VATS (versus lobectomy by VATS) VATS: Video-assisted thoracic surgery, OR: Odds ratio, CI: Confidence interval, COPD: Chronic obstructive pulmonary disease. [file 13019_2019_1021_MOESM1_ESM.docx]

Additional file 1. Multivariate logistic regression models to predict segmentectomy by VATS (versus lobectomy by VATS)

| Variables | OR (95% CI) | P |
| --- | --- | --- |
| Woman (vs man) | 1.56 (1.10 – 2.22) | 0.012 |
| COPD (vs no COPD) | 1.77 (1.24 – 2.54) | 0.002 |
| Histology  Lung cancer (primary)  Metastasis  Benign lesion  Emphysema  Infection | 1 (reference)  6.06 (2.84 – 12.93)  5.42 (2.21 – 13.31)  0 (not estimable)  2.45 (0.93 – 6.44) | <0.001  <0.001  <0.001  -  0.069 |
| Location (lobe)  Upper right  Middle right  Lower right  2 right lobes  Upper left  Lower left | 1 (reference)  0.15 (0.03 – 0.68)  2.12 (1.26 – 3.57)  0 (not estimable)  2.95 (1.83 – 4.75)  4.58 (2.76 – 7.60) | <0.001  0.014  0.005  -  <0.001  <0.001 |
| Nagelkerke R^2^ | 0.230 | |

VATS: Video-assisted thoracic surgery, OR: Odds ratio, CI: Confidence interval, COPD: Chronic obstructive pulmonary disease.
