# Supplementary material for: Comparison of postoperative complications between segmentectomy and lobectomy by video-assisted thoracic surgery: a multicenter study
Source: J Cardiothorac Surg. 2019 Nov 7;14:189. doi: 10.1186/s13019-019-1021-9 (PMC6836384; doi:10.1186/s13019-019-1021-9)
Supplement: Supplementary file 2 — Additional file 2. Comparison of propensity score according to type of intervention by VATS VATS: Video-assisted thoracic surgery, SD: Standard deviation, AUC: Area under the curve, CI: Confidence interval. [file 13019_2019_1021_MOESM2_ESM.docx]

Additional file 2. Comparison of propensity score according to type of intervention by VATS

|  | Segmentectomy | Lobectomy | AUC (95% CI) |
| --- | --- | --- | --- |
| Propensity (probability of segmentectomy), mean (SD) | 0.46 (0.19) | 0.29 (0.17) | 0.744  (0.706 – 0.782) |
| Quintiles, N (%)  1  2  3  4  5 | 10 (4.2)  29 (12.1)  49 (20.4)  58 (24.2)  94 (39.2) | 97 (21.6)  117 (26.0)  107 (23.8)  84 (18.7)  45 (10.0) | 0.735  (0.696 – 0.773) |

VATS: Video-assisted thoracic surgery, SD: Standard deviation, AUC: Area under the curve, CI: Confidence interval.
