# Supplementary material for: Comparison of postoperative complications between segmentectomy and lobectomy by video-assisted thoracic surgery: a multicenter study
Source: J Cardiothorac Surg. 2019 Nov 7;14:189. doi: 10.1186/s13019-019-1021-9 (PMC6836384; doi:10.1186/s13019-019-1021-9)
Supplement: Supplementary file 3 — Additional file 3. Associations of patient characteristics with complications. VATS: Video-assisted thoracic surgery, ASA: American Society of Anesthesiologists Score, BMI: Body mass index, PY: pack-years, COPD: Chronic obstructive pulmonary disease, FEV1: Forced expiratory volume in one second, DLCO: Diffusing capacity of the lungs for carbon monoxide, SLND: systematic lymph node dissection. [file 13019_2019_1021_MOESM3_ESM.docx]

Additional file 3. Associations of patient characteristics with complications

| Characteristics | N (%) | Risk of complication  N=240 | P |
| --- | --- | --- | --- |
| Intervention  VATS lobectomy  VATS segmentectomy | 450 (65.2)  240 (34.8) | 38.0%  33.3% | 0.24 |
| Center  1  2 | 432 (62.6)  258 (37.4) | 39.4%  31.4% | 0.041 |
| Gender  Man  Woman | 375 (54.3)  315 (45.7) | 38.9%  33.3% | 0.13 |
| Age  21-59  60-69  70-79  80-90 | 168 (24.3)  248 (35.9)  215 (31.2)  59 (8.6) | 31.0%  38.4%  41.4%  25.4% | 0.047 |
| ASA score  1  2  3  4 | 6 (0.9)  377 (54.6)  295 (42.8)  8 (1.2) | 66.7%  29.4%  44.1%  50.0% | <0.001 |
| BMI (mg/kg^2^)  14.5-18.4  18.5-24.9  25-29.9  30.0-57.0 | 46 (6.7)  305 (44.2)  223 (32.3)  110 (15.9) | 45.7%  36.7%  33.6%  35.5% | 0.48 |
| Active smoker  No  Yes | 396 (57.4)  293 (42.5) | 34.8%  38.2% | 0.38 |
| PY, 117 missing  0  1-49  50-160 | 150 (26.2)  219 (38.2)  204 (35.6) | 21.5%  34.1%  44.4% | 0.004 |
| High blood pressure  No  Yes | 355 (51.4)  345 (48.6) | 33.0%  40.0% | 0.058 |
| Cardiopathy  No  Yes | 602 (87.2)  88 (12.8) | 36.0%  38.6% | 0.64 |
| Diabetes  No  Yes | 586 (84.9)  102 (14.8) | 36.3%  37.3% | 0.91 |
| COPD  No  Yes | 431 (62.5)  259 (37.5) | 31.8%  44.0% | 0.001 |
| History of cancer  No  Yes | 461 (66.8)  229 (33.2) | 37.7%  33.6% | 0.31 |
| FEV1 (%), 28 missing  24-59  60-79  80-170 | 53 (8.0)  169 (25.5)  440 (66.5) | 52.8%  45.0%  31.6% | <0.001 |
| DLCO (%), 54 missing  26-60  61-80  81-154 | 125 (19.7)  238 (37.4)  273 (42.9) | 46.4%  37.8%  31.1% | 0.012 |
| Indication  Lung cancer (primary)  Metastasis  Benign lesion  Emphysema  Infection | 596 (86.4)  40 (5.8)  27 (3.9)  5 (0.7)  22 (3.2) | 38.9%  15.0%  11.1%  60.0%  31.8% | 0.001 |
| Side  Right  Left | 375 (54.3)  315 (45.7) | 37.9%  34.6% | 0.38 |
| Location (lobe)  Upper right  Middle right  Lower right  2 right lobes  Upper left  Lower left | 198 (28.7)  38 (5.5)  128 (18.6)  11 (1.6)  180 (26.1)  135 (19.6) | 40.4%  15.8%  39.1%  54.5%  35.0%  34.1% | 0.056 |
| SLND  No  Yes | 76 (11.0)  614 (89.0) | 38.2%  36.2% | 0.80 |

VATS: Video-assisted thoracic surgery, ASA: American Society of Anesthesiologists Score, BMI: Body mass index, PY: pack-years, COPD: Chronic obstructive pulmonary disease, FEV1: Forced expiratory volume in one second, DLCO: Diffusing capacity of the lungs for carbon monoxide, SLND: systematic lymph node dissection.
